# Design strategies for flexible core biopsy needles: insights from patent literature

**DOI:** 10.3389/fmedt.2026.1771655

**Published:** 2026-04-01

**Authors:** Nynke M. Ansems, Dalibor Vasilic, Paul Breedveld

**Affiliations:** 1 Department of Plastic and Reconstructive Surgery, Erasmus MC University Medical Center, Rotterdam, Netherlands; 2 Department of BioMechanical Engineering, Faculty of Mechanical Engineering, Delft University of Technology, Delft, Netherlands

**Keywords:** biopsy needle tip, catheter-based biopsy, core biopsy, endoscopic biopsy, flexible biopsy needle, medical device design, minimally invasive cancer diagnostics, patent analysis

## Abstract

Flexible biopsy needles are essential for minimally invasive cancer diagnostics, enabling core biopsy sampling through endoscopic and catheter-based procedures. These approaches allow clinicians to access deep-seated or anatomically challenging lesions while reducing patient risk compared to traditional percutaneous methods. However, the shift toward minimally invasive techniques imposes competing design demands on the biopsy needle tip, which must be miniaturized and flexible enough to navigate narrow lumens while maintaining cutting efficiency and retaining adequate tissue samples for clinical diagnosis. This review presents a patent-based analysis of flexible core biopsy needle designs, focusing on mechanisms at the needle tip that govern tissue cutting and retention. We systematically evaluated 65 international patent applications and developed a classification framework for cutting and gripping strategies. Using this framework, we identified dominant technological trends and highlighted underexplored concepts. Forward-cutting tips combined with suction or macroshape grips that engage the sample from the front dominate current designs due to their mechanical simplicity. However, alternative approaches such as compliant tip designs and multidirectional cutting mechanisms offer opportunities to enhance tissue yield without increasing device diameter. Implementing these innovations could reduce the need for repeated insertions, improve sampling efficiency, and enable access to lesions previously considered unreachable. This review provides an overview of flexible core biopsy needle designs and offers insights to guide future innovations that advance minimally invasive cancer diagnostics.

## 1 Introduction

Clinicians diagnose a wide range of malignancies by obtaining tissue samples from suspicious lesions, a procedure known as biopsy (1). While full excision was once the standard diagnostic approach, current practice increasingly relies on minimally invasive biopsy techniques (2). One widely used method is percutaneous core needle biopsy (CNB), in which a biopsy needle is inserted directly through the skin, sometimes passing through other organs, to reach the lesion (1, 3). CNB aims to obtain a solid tissue core that can be used for histological analysis (2). Although advanced imaging systems assist in planning and guiding safe needle trajectories, certain lesions remain inaccessible due to the absence of a safe percutaneous route. In such cases, the inability to perform a biopsy can lead to diagnostic uncertainty and delays in initiating personalized treatment (2, 4).

To overcome this limitation, several alternative biopsy techniques have been developed. In an operative setting, laparoscopic ultrasound-guided biopsy can be performed with instruments introduced through a laparoscope working channel (5). Although effective, this approach requires surgical access. Flexible biopsy needles provide a fundamentally different strategy for reaching deep-seated lesions. By navigating the body's natural luminal pathways, such as the gastrointestinal (GI) tract, bronchial tree, or blood vessels, they can access targets adjacent to these lumens. By using these internal routes, flexible biopsy needles offer potentially safer and more direct access to otherwise unreachable targets.

**Figure 1:**
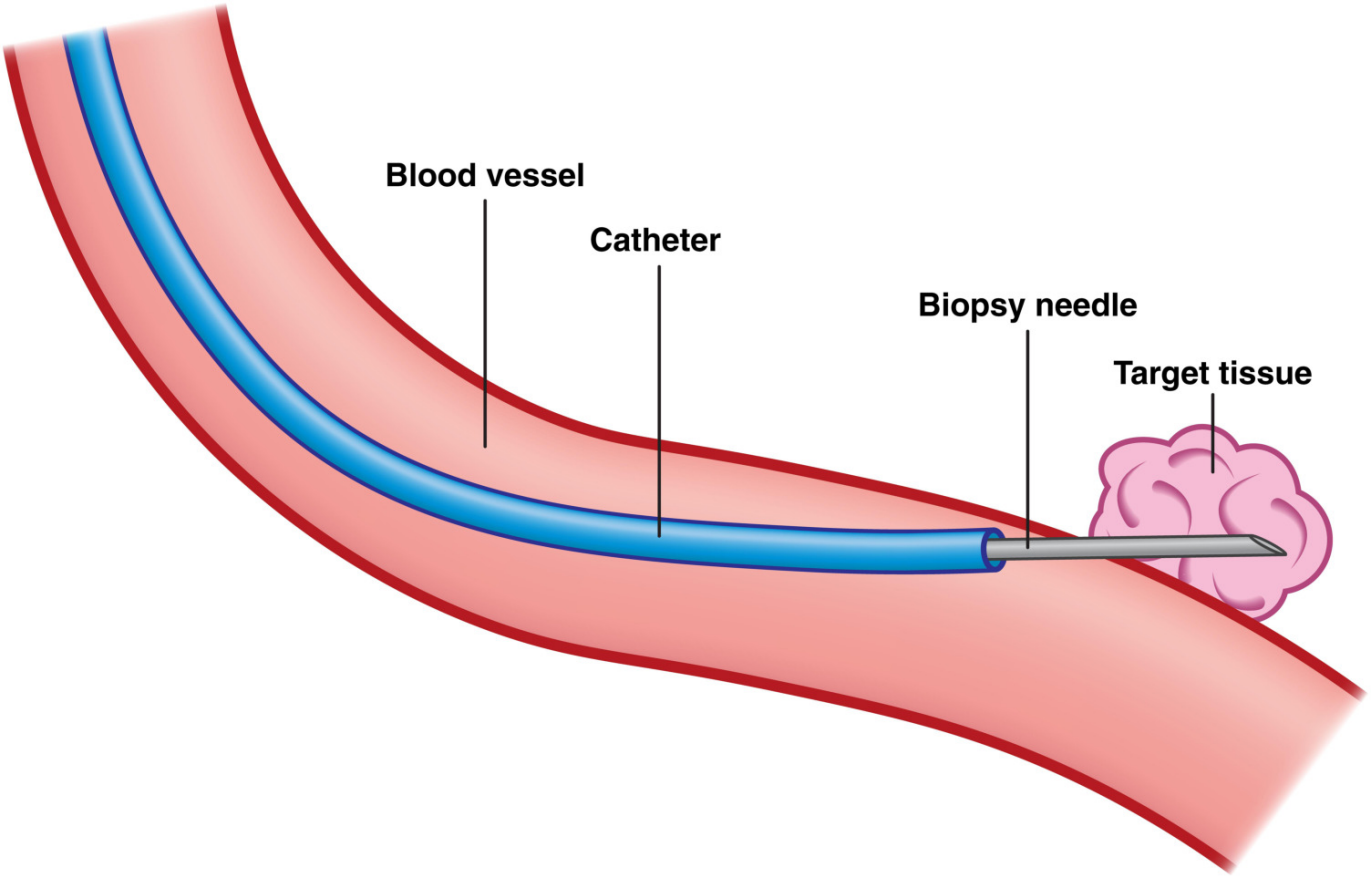
Schematic representation of a biopsy procedure using a flexible biopsy needle (grey) inserted through a catheter (blue), which is navigated via the blood vessel (red) to sample the target tissue (pink) located adjacent to the vessel.

Two primary access routes are used to introduce flexible biopsy needles into the body: (1) via natural orifices, such as the mouth, using the working channel of an endoscope, or (2) percutaneously through the skin to reach blood vessels using a catheter. Once introduced, the needle is navigated through the corresponding luminal pathway to the target site, where it penetrates the luminal wall to access adjacent tissue and retrieve a core tissue sample (see Figure 1).

Each access route imposes distinct anatomical constraints, necessitating specialized introducer instruments and imaging modalities to guide the needle accurately. Among the most established techniques are endoscopic ultrasound (EUS)-guided and endobronchial ultrasound (EBUS)-guided biopsies, which enable sampling of solid lesions adjacent to the GI tract and airways, respectively (6, 7). Both techniques employ a flexible endoscope equipped with an ultrasound probe, allowing a biopsy needle to be introduced through the endoscope's working channel.

Beyond the GI and respiratory tract, blood vessels offer an alternative route to deep-seated lesions. Transvenous biopsy (TVB) has traditionally been used for random liver biopsies under fluoroscopic guidance (8). More recently, researchers have explored TVB for sampling abdominal and pelvic lymph nodes, integrating a transjugular liver biopsy needle with real-time imaging from an intracardiac echocardiography catheter (9, 10). While these clinical studies drive the exploration of novel biopsy procedures, their diagnostic success depends on corresponding innovations in the biopsy needle design.

The design of flexible core biopsy needles for endoscopic and catheter-based procedures involves a complex balance of mechanical and clinical requirements. To reach deep-seated lesions through complex luminal pathways, the needle shaft must be sufficiently flexible to navigate often torturous anatomical structures. However, this flexibility reduces the efficiency of the force transmission from the device handle to the needle tip, which is important for effective tissue cutting. To further improve maneuverability within narrow body lumens, thin needles are preferred, typically ranging from 0.72 to 1.10 mm in diameter (19 to 25 gauge) (8, 11). Despite these mechanical limitations, the needle must still retrieve a tissue core of sufficient volume for histopathological and molecular analysis (12). Sample requirements vary by clinical context, ranging from 5 mm^3^ for lymph nodes to 9 mm^3^ for lung and colon lesions (4). The competing demands of miniaturization, flexibility, and sample adequacy place particular design demands on the needle tip, which must cut tissue effectively and retain reliable samples for clinical diagnosis.

To address these challenges, multiple generations of flexible biopsy needles have been developed, featuring a range of innovative tip designs (6, 13). Much of this innovation has focused on fine needle biopsy (FNB) devices. These are flexible biopsy needles designed to obtain a solid tissue core, similar to CNB, but intended for endoscopic use (6). Among these devices, newer end-cutting FNB needles have demonstrated improved diagnostic accuracy as compared to earlier models (11). Nevertheless, to obtain adequate tissue samples from a single lesion, current FNB needles often require multiple reinsertions, as well as repeated back-and-forth movements during a single insertion (11). These limitations highlight the need for continued innovation in flexible biopsy needle design to enhance sampling efficiency in endoscopic and catheter-based biopsy procedures.

Although the clinical performance of FNB needles is extensively studied, comparative studies and review papers typically focus on commercially available products, often within the scope of a single pathology (6, 14). As a result, these studies offer limited insight into the design rationale behind various biopsy needle tips, narrowing our understanding of broader technological trends and providing little guidance for future device development.

The goal of this review is to provide a structured overview of patent literature on flexible biopsy needles for endoscopic and catheter-based procedures, with a focus on the biopsy mechanism at the needle tip for obtaining core tissue samples. By analyzing design strategies, we aim to identify technological trends and highlight promising directions for future innovation. Patent literature was selected as the primary source because it offers early insight into emerging technologies and design concepts that are often not yet reflected in clinical studies.

## 2 Methods

### 2.1 Patent search method

We conducted a patent search using the Espacenet patent database. Unlike scientific literature searches, which typically rely on keyword-based queries, patent searches are more effectively performed using patent classification codes. These codes reflect the technical content of a patent and are assigned by patent examiners. Every patent filed with a member of the World Intellectual Property Organization (WIPO) receives at least one classification code from the International Patent Classification (IPC) system. This system is hierarchical, meaning that IPC codes range from broad categories to highly specific subcategories. To ensure precision, patents are assigned the most detailed classification codes available.

Based on this approach, we began our search with IPC code A61B10/02, which broadly covers instruments used for cell sampling and biopsy. To narrow the focus, we selected patents that were simultaneously classified under two more specific subcodes: A61B10/0233 and A61B10/04, including all their respective subcategories. A61B10/0233 pertains to “pointed or sharp biopsy instruments”, and A61B10/04 covers “endoscopic instruments e.g., catheter-type devices”. By combining these two subcodes, our search specifically targeted needle-like biopsy instruments designed for endoscopic and catheter-based procedures.

To refine the results, we applied filters to include only patents filed under the WIPO, as these are considered to represent key technological developments in the field. We also limited the search to patents available in English to ensure they were readily accessible for analysis. No restrictions were placed on publication date, to ensure a comprehensive overview of technological developments in the field. The final Espacenet query used was: (cl="A61B10/0233/low”) AND (cl="A61B10/04/low”), with filters applied for “ Countries (publication): WO” and “Languages (publication): en”.

### 2.2 Eligibility criteria

A patent was included in this review only if it met all of the following criteria. First, it needed to describe an innovation at the needle tip related to the biopsy mechanism. Patents that focused exclusively on other components, such as the shaft, handle, or visualization of the tip, were excluded. Second, the biopsy mechanism had to be intended for retrieving a core tissue sample, rather than being limited to fluid aspiration or cell sampling. Third, the mechanism had to be compatible with a fully flexible shaft and designed for use in endoscopic or catheter-based procedures. Biopsy mechanisms intended solely for rigid shafts did not meet the inclusion criteria. Finally, the device had to be intended for sampling tissue located beyond the lumen of the organ used for navigation e.g., a lymph node adjacent to the airways. Devices designed only to sample the lumen wall were excluded, as they are suited only for superficial sampling, not for accessing deep-seated lesions beyond the lumen.

### 2.3 Patent search results

The patent search yielded a total of 458 patents (December 2025). During the initial screening, the title, abstract, and drawings of each patent were reviewed according to the eligibility criteria. This initial screening identified 131 potentially relevant patents. Subsequently, the full-text of these patents was examined, including a detailed analysis of their claims, descriptions, and drawings. Following this in-depth evaluation, 66 patents were excluded, leading to a final selection of 65 relevant patents included in this review. An overview of these patents is provided in Supplementary Table S1.

### 2.4 Classification

**Figure 2:**
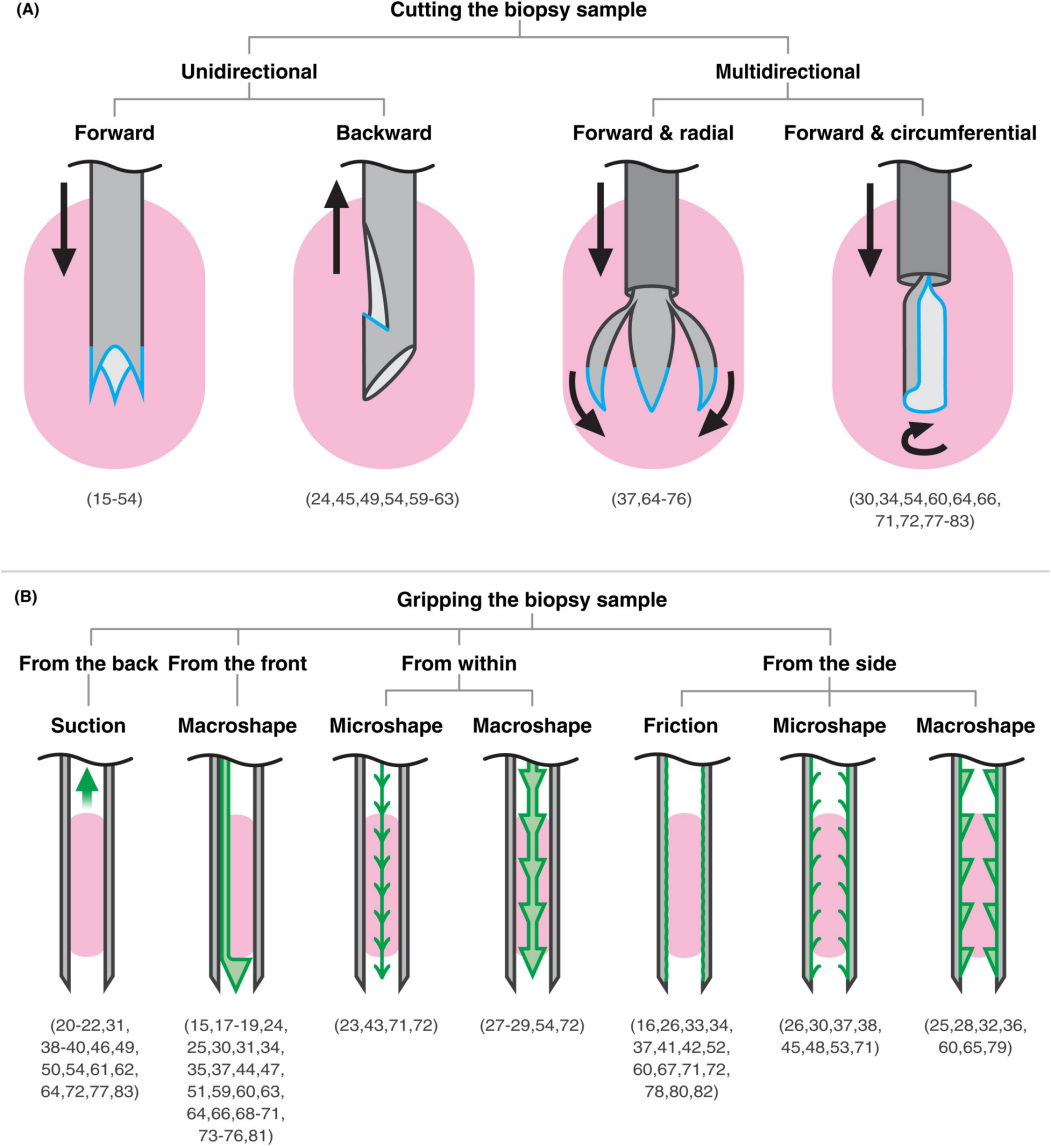
Classification of biopsy needle designs (grey: needle body, blue: cutting edges, green: gripping mechanisms, pink: tissue). **(A)** Cutting mechanisms by direction of motion separating the sample from surrounding tissue. **(B)** Gripping mechanisms by grip location relative to the sample and type of grip. References are listed in brackets under each group. Patents with multiple strategies appear in more than one group.

Rather than presenting the patents individually, we organized them into a classification framework that highlights common design strategies and serves as the outline for the body of this review (see Figure 2). The included patents were classified according to how the needle cuts and grips the biopsy sample.

Cutting mechanisms were categorized by the direction of the cutting motion that separates the sample from surrounding tissue. This classification excludes the initial puncture of the needle to reach the lesion and focuses solely on the detachment of the sample. The first group, “unidirectional” cutting, employs an axial cutting motion strictly along the insertion axis. These mechanisms fall into two subgroups: “forward” cutting (during insertion) and “backward” cutting (during retraction). In contrast, “multidirectional” cutting combines forward motion with cutting in an additional plane. This secondary plane may be “radial”, moving inward or outward from the insertion axis, or “circumferential”, rotating around the insertion axis.

Gripping mechanisms were classified based on both the location of grip relative to the biopsy sample and the type of grip applied. Gripping serves three primary functions: stabilizing tissue before and during cutting to counteract tissue displacement, securing the sample within the needle after cutting, and facilitating complete detachment of the sample during needle retraction. The first classification level distinguishes grip applied “from the back”, “from the front”, “from within”, or “from the side” of the biopsy sample, where “front” refers to the distal end of the needle. The second classification level specifies the type of grip: “suction”, “friction”, “microshape”, or “macroshape”. Friction-based grip relies solely on surface contact between the sample and the needle wall. Microshape grip employs small features that superficially interlock with the sample, whereas macroshape grip uses larger structures that engage the sample more securely.

The distinction between micro- and macroshape grips was based on the relative size of the gripping features as illustrated in the patent drawings. Features depicted as individually protruding to less than approximately one quarter of the needle lumen diameter were classified as microshape, whereas features exceeding this relative size were classified as macroshape. Because patent drawings are not always to scale and often lack explicit dimensional information, these boundaries are not always clear. Classifications were therefore made to the best of our judgment. All patents were initially classified by the first author, and borderline or ambiguous cases were discussed with the last author until consensus was reached.

Several patents describe multiple distinct designs employing different cutting or gripping strategies, or a single design that combines multiple approaches. Consequently, a single patent could be assigned to more than one group within our classification framework. Each group will be discussed in detail in the following sections, outlining the general design principle and highlighting different specific design approaches found in the included patents, accompanied by illustrative example figures.

## 3 Cutting the biopsy sample

**Figure 3:**
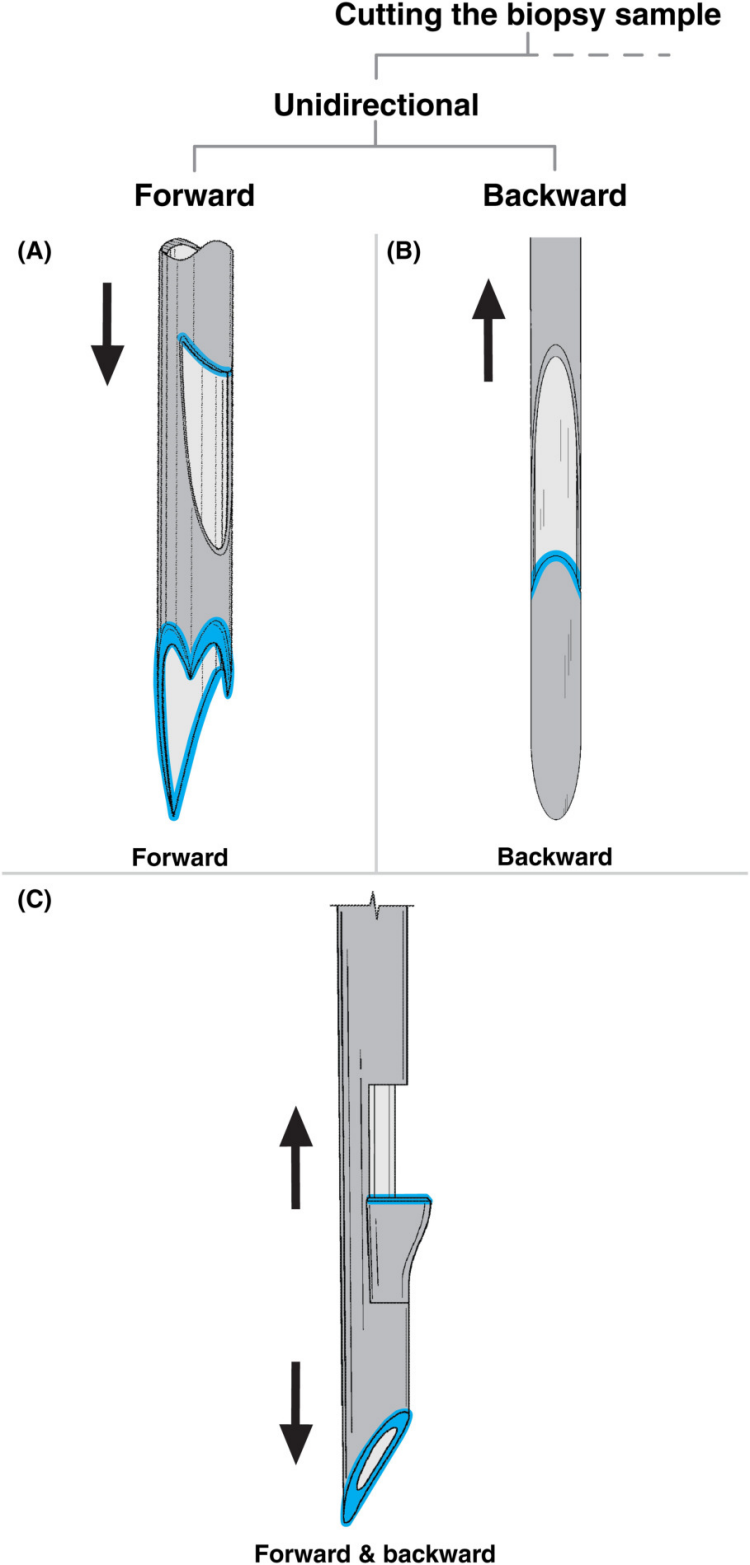
Biopsy needles included in this review, highlighting unidirectional cutting mechanisms (grey: needle body, blue: cutting edge, arrows indicate direction of cutting motion). **(A)** Forward-cutting needle with a multi-point serrated distal end and a side opening, adapted from (50). **(B)** Backward-cutting needle with a side opening, adapted from (62). **(C)** Needle combining forward and backward cutting via a distal-end opening and side opening, adapted from (54).

**Figure 4:**
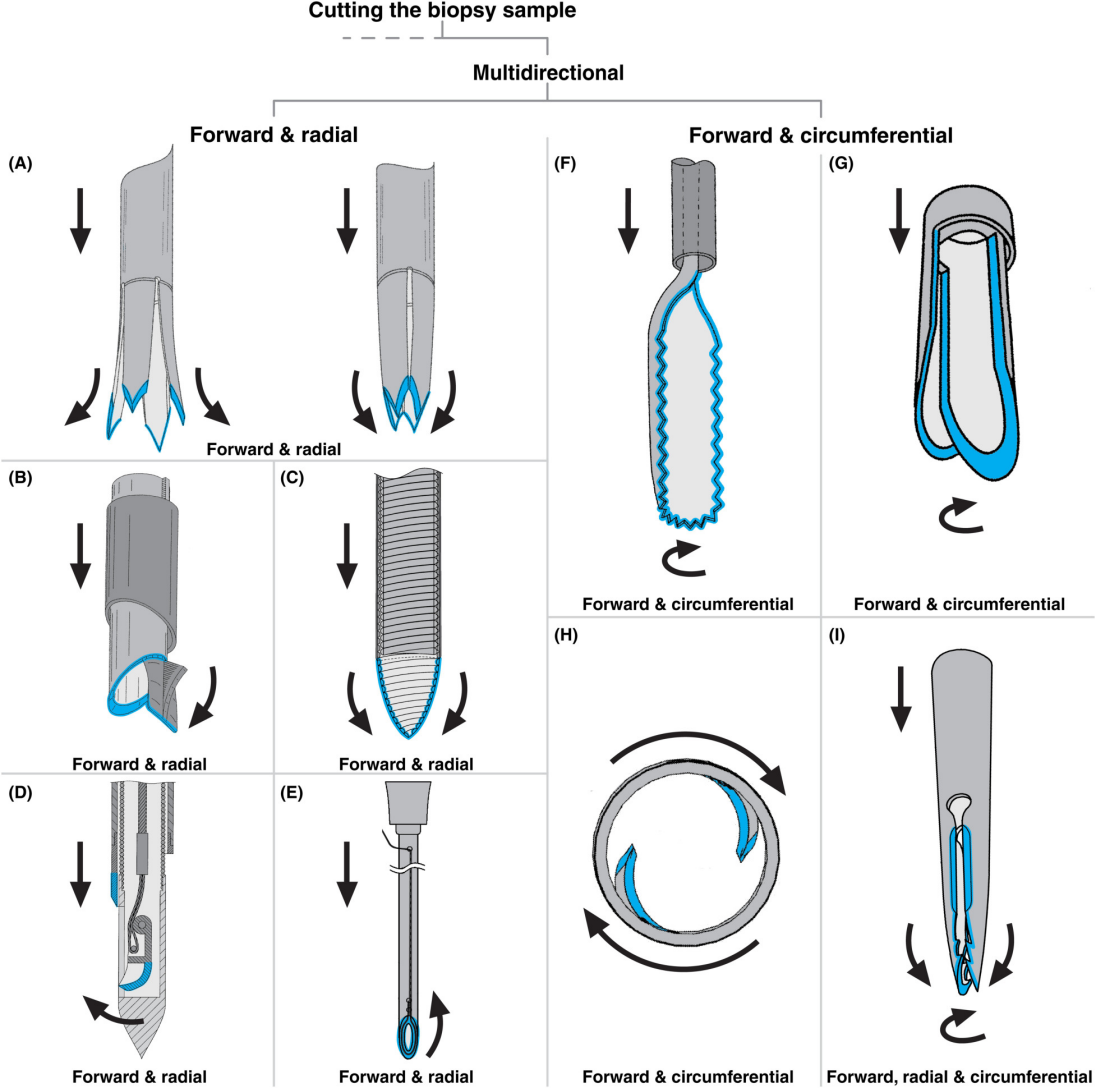
Biopsy needles included in this review, highlighting multidirectional cutting mechanisms (grey: needle body, blue: cutting edge, arrows indicate direction of cutting motion). **(A)** Forward- and radial-cutting needle with a compliant split tip that opens and closes in response to tissue pressure, adapted from (69). **(B)** Forward- and radial-cutting needle featuring a cam mechanism actuated by the outer cannula, adapted from (75). **(C)** Forward- and radial-cutting needle with a conical, inward-biased tip that opens upon forward rotation of the inner cannula, adapated from (68). **(D)** Forward- and radial-cutting needle with a rigid, hinged cutting segment that rotates radially outward about a linking pin, adapted from (76). **(E)** Forward- and radial-cutting needle incorporating a cutting thread routed along the cutting edge, which cuts radially inward when pulled proximally, adapted from (67). **(F)** Forward- and circumferential-cutting needle in which the outer cannula advances to close an inner compliant, serrated needle tip, adapted from (71). **(G)** Forward- and circumferential-cutting needle with two coaxial cutting cannulas operating in a scissor-like manner, adapted from (34). **(H)** Forward- and circumferential-cutting needle incorporating U-shaped cut-outs that form compliant cutting segments, adapted from (81). **(I)** Forward-, radial-, and circumferential-cutting needle in which a split tip cuts radially inward, while longitudinal slit edges cut during rotation about the insertion axis, adapted from (64).

During endoscopic or catheter-based biopsy procedures, the flexible biopsy needle is typically guided through the working channel of an endoscope or a catheter with a stiff distal end. Tissue access is achieved by advancing the needle into the lesion. The following sections focus on the mechanisms used to cut the biopsy sample, excluding the initial step of lesion access. Representative examples of each cutting strategy are presented in Figures 3, 4. In addition, two examples are highlighted that combine cutting mechanisms from multiple groups.

### 3.1 Unidirectional cutting

#### 3.1.1 Forward cutting

Within the unidirectional cutting group, a total of 40 patents (15–54) describe biopsy needles that sever tissue by advancing forward along the insertion axis. This forward motion is generated either manually or via a spring-based actuation system located in the device handle outside the patient's body. The spring mechanism propels the cutting needle tip rapidly, minimizing tissue deformation during cutting and thereby increasing the sample volume (55, 56).

The most common forward-cutting needle designs feature a hollow cannula with an open distal end and a sharpened cutting edge (15–44, 46–48, 50–54). As the cannula advances, tissue enters through the distal opening, while the cutting edge severs the sample from the surrounding tissue. The simplest variants employ a continuous circular cutting edge, with the distal opening either flat-ended or beveled.

More complex variants are described in eight patents (16, 21, 33, 39–41, 46, 50), which specifically focus on optimizing the geometry of the cutting edge to reduce cutting force. A lower cutting force is expected to minimize tissue deformation and improve both sample volume and histological quality (57). These designs often divide the distal end into multiple sharp points connected by beveled cutting edges. The points may be of equal length (21, 33, 41) or designed with one point longer than the others (see Figure 3A) (39, 46, 50). Multi-point serrated designs, such as the one in Figure 3A, distribute the cutting force across several points, which may reduce tissue deformation during cutting. Additionally, the rake and inclination angles (58) of the cutting edges are optimized to further reduce cutting force. One patent focuses exclusively on refining the bevel geometry of a single, multi-faceted needle tip to enhance cutting performance (40).

In contrast to distal-end cutting designs, five patents (24, 39, 45, 49, 50) describe biopsy needles with one or more side openings in a (partially) hollow cannula, where the proximal edge of the opening is sharpened to form a cutting edge (see Figure 3A). Once the needle reaches the target site, tissue enters through the side opening. As the cannula advances forward, the proximal cutting edge severs the sample from the surrounding tissue. Two patents integrate both distal and side cutting openings within a single biopsy needle, as shown in Figure 3A (39, 50).

#### 3.1.2 Backward cutting

Nine patents (24, 45, 49, 54, 59–63) within the unidirectional cutting group describe biopsy needles that cut tissue while moving backward along the insertion axis (during retraction). The most common backward-cutting needle design resembles the forward-cutting needle with a side opening. It also consists of a (partially) hollow cannula featuring an opening on the side, as illustrated in Figure 3B. In contrast to the forward-cutting variant, however, the distal edge of the side opening serves as the cutting edge. Once the needle reaches the target tissue, tissue enters through the side opening. As the needle retracts, the distal cutting edge severs the collected tissue.

Two variations of this design were identified. Five patents (45, 49, 54, 60, 62) describe a single-cannula design, as outlined in the previous paragraph. In contrast, three patents (24, 60, 61) incorporate an additional outer cannula. In this dual-cannula design, the inner cannula retracts within the outer cannula during cutting, thereby closing the side opening. The outer cannula may also stabilize the tissue during cutting, potentially enhancing sampling efficiency.

**Figure 5:**
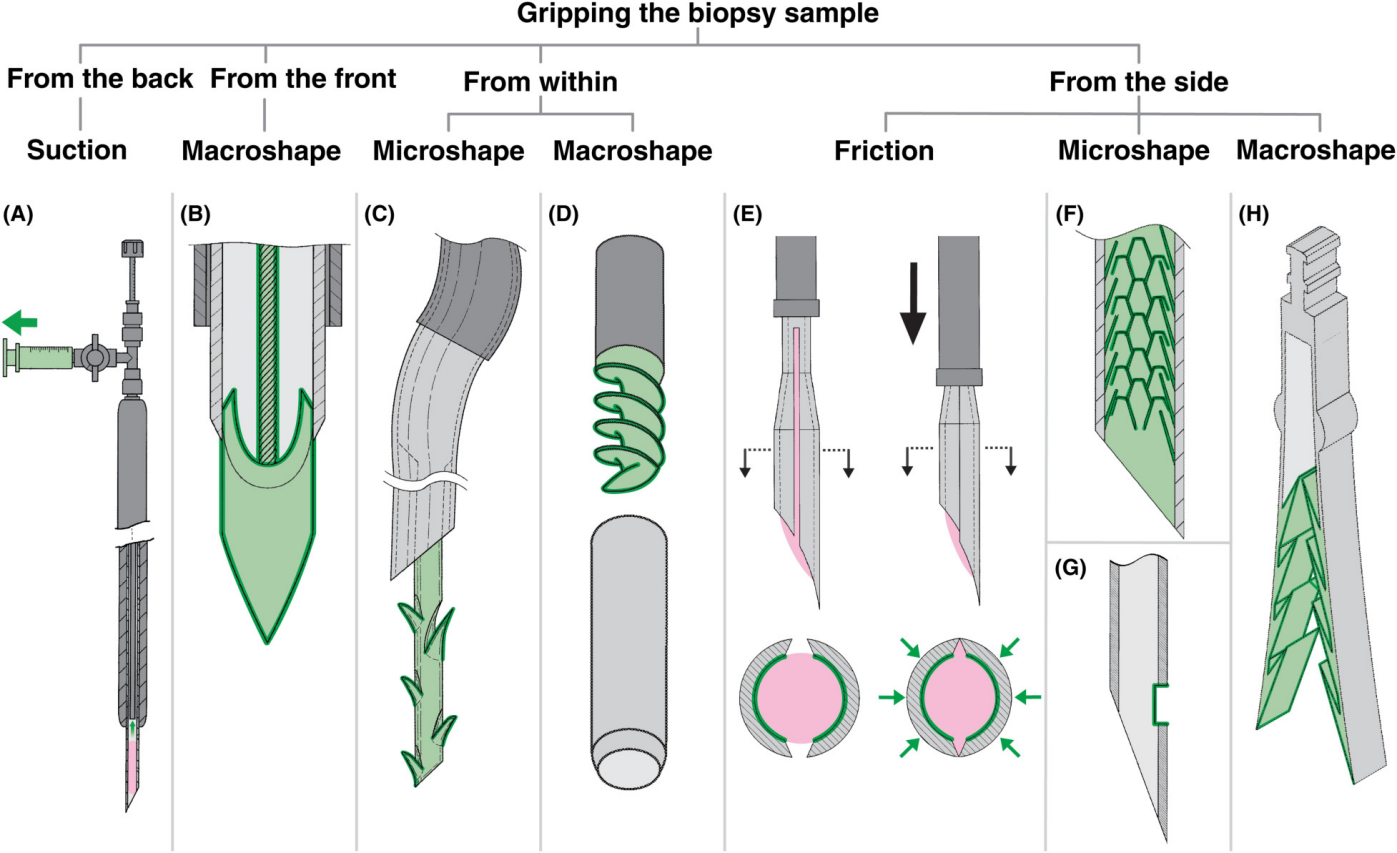
Biopsy needles included in this review, highlighting gripping mechanisms (grey: needle body, green: gripping mechanism, pink: tissue). **(A)** Needle employing suction to provide grip from the back of the sample using a syringe, adapted from (22). **(B)** Needle providing macroshape grip from the front of the sample with a backward-cutting piercing tip, adapted from (44). **(C)** Needle using an anchoring thread with microbarbs to provide microshape grip from within the sample, adapted from (23). **(D)** Needle using a screw to provide macroshape grip from within the sample, adapted from (29). **(E)** Compliant, expandable needle that increases friction from the side of the sample through compression, adapted from (80). **(F)** Needle with an inner scaled surface to provide microshape grip from the side, adapted from (37). **(G)** Needle with a small opening that allows the sample to bulge inward, providing microshape grip from the side, adapted from (53). **(H)** Needle with inward-facing hooks providing macroshape grip from the side, adapted from (65).

A distinctly different backward-cutting design comprises a hollow cannula with a distal opening and a sharp tip mounted on an actuating rod running through the cannula (59, 63). Although Figure 5B is discussed in Section 4.2.1 on macroshape grip from the front, it provides a clear illustration of this design. The tip, which matches the diameter of the cannula lumen, features sharpened proximal edges that act as cutting surfaces. During sampling, the tip extends beyond the cannula to penetrate the tissue. As it retracts, tissue is trapped between the tip and the cannula wall and severed by the cutting edges of the tip. The sample is then enclosed within the hollow cannula. Similar to the previously described dual-cannula design, the cannula may provide counterforce to improve cutting efficiency.

#### 3.1.3 Forward & backward cutting

While the previous sections described biopsy needles that rely exclusively on either forward or backward motion for sample cutting, some designs integrate both strategies within a single device. Three patents (24, 49, 54) describe such hybrid needles, which combine forward and backward cutting mechanisms without introducing structural features beyond those outlined in Sections 3.1.1 and 3.1.2.

As illustrated in Figure 3C, Shaubhut et al. (54) present a design that initially cuts tissue via the distal-end opening during insertion. During retraction, a compliant, expandable distal cutting blade located within a side opening severs the tissue. In contrast, two patents (24, 49) describe a hollow cannula with a side opening featuring sharpened proximal and distal edges. This configuration enables tissue cutting during both forward and backward motion through the same side opening.

### 3.2 Multidirectional cutting

#### 3.2.1 Forward & radial cutting

Among multidirectional cutting biopsy needles, fourteen patents (37, 64–76) describe designs that combine forward and radial motion, cutting either inward or outward from the insertion axis. Radially inward cutting can (partially) sever the tissue at the front of the sample, increasing the likelihood that the sample remains inside the needle during retraction, as it becomes fully detached from the surrounding tissue. In contrast, radially outward cutting needles typically aim to obtain a larger sample than a simple forward-cutting needle by temporarily expanding the needle lumen.

The patent literature reveals several approaches to achieving a combined forward and radial cutting motion. The most frequently described method employs compliant hinged cutting segments. A compliant hinge is a flexible segment that is designed to bend under an applied force without breaking, typically fabricated from superelastic materials such as nickel-titanium alloys. For example, Kadamus et al. (69) present a needle tip consisting of a hollow cannula with a multi-tooth distal end (see Figure 4A). The tip is divided into multiple teeth separated by V-shaped cutouts, each transitioning into a longitudinal slit extending proximally along the shaft. During insertion, tissue reaction forces deflect the teeth radially outward, increasing the lumen's capacity to capture tissue. Upon retraction, Kadamus et al. claim that the teeth collapse inward, partially severing tissue radially while simultaneously compressing the sample. However, the mechanism by which this inward motion is generated solely by tissue pressure remains unclear to the authors of this paper. Two other patents describe similar designs in which tissue reaction forces govern the radial movement of the needle tip (64, 70).

In five other patents (65, 71, 72, 74), compliant hinged segments are actuated by an outer cannula rather than tissue forces. In these designs, the cutting segments are biased outward but are constrained by the outer cannula. During sampling, the outer cannula advances over the compliant cutting segments, forcing them to bend inward, as shown in the schematic drawing of a forward & radial cutting biopsy needle in Figure 2A. Gordon (75) presents a variation of this design in which the cutting segment is initially straight rather than biased outward (see Figure 4B). A cam mechanism on top of the segment is activated as the outer cannula slides forward, bending the segment inward to achieve radial cutting.

A unique compliant design is presented by Vetter & Vetter (68), involving a two-piece needle in which the inner cannula provides the force to counteract the radially inward bending of the outer cannula. The outer cannula consists of helical windings with a tip biased inward, forming a sharp conical point (see Figure 4C). The inner cannula, featuring external helical windings, is straight. When rotated and advanced, the inner cannula opens the conical tip, allowing tissue entry. When rotated backward, the outer cannula closes, cutting tissue via inward radial motion.

In addition to tissue and outer-cannula actuation, two patents describe needle designs with compliant cutting segments made from a shape-memory metal that is actuated by temperature changes (64, 73). For example, Shang (73) presents a needle tip similar to the one shown in Figure 4A, designed for use in blood vessels. At body temperature (37°C), the tip remains open, with the cutting segments aligned with the rest of the cannula. When a cooled saline solution (5°C) is injected through the needle lumen, the segments close radially inward, enabling radial cutting. However, the injected solution could potentially flush out the biopsy sample.

In contrast to compliant designs, three patents (37, 66, 76) describe a needle with rigidly hinged cutting segments that rotate radially inward or outward around a linking pin, typically actuated by a push–pull rod (see Figure 4D). These mechanisms require the fabrication of multiple miniature components, posing severe and potentially very expensive miniaturization challenges at the small scales required for such devices.

One patent presents a completely different approach to radial cutting. Ueki and Matsumoto (67) describe a biopsy needle formed from a hollow cannula with a beveled distal opening. On the short side of the bevel, two small longitudinal holes are positioned in the cannula wall. A cutting thread is anchored through these holes and routed along the beveled cutting edge in a loop, held in place by an easy-peel adhesive (see Figure 4E). The free end of the thread passes through the needle lumen and exits at the proximal end of the device. During sampling, the needle is first inserted into the target tissue, initially cutting with a forward motion. Once positioned, the operator pulls the free end of the thread, releasing the loop from the cutting edge and tightening it across the beveled opening. As the loop constricts, the thread severs the tissue along the bevel in a radially inward motion.

#### 3.2.2 Forward & circumferential cutting

Fifteen patents (30, 34, 54, 60, 64, 66, 71, 72, 77–83) describe biopsy needles that integrate forward and circumferential cutting, wherein a cutting blade rotates around the insertion axis. The patent literature outlines several methods for achieving this combined cutting motion.

A common a design approach involves a needle composed of a straight, rigid outer cannula and a compliant inner cannula featuring one or more longitudinal slits extending from its distal end (see Figure 4F) (30, 71, 72, 78, 80). These slits create compliant segments that expand outward from the lumen of the outer cannula, resembling an unfolding rolled-up sheet. The sharpened edges of the slits serve as cutting surfaces. During sampling, the expanded inner cannula first cuts tissue in a forward direction. As the outer cannula advances, it compresses the inner cannula back into its rolled-up form, driving the slit edges to cut circumferentially while simultaneously compressing the sample.

An alternative approach (60, 64, 77, 82, 83) employs a rigid hollow cannula with an open distal end and one or more longitudinal side openings. These openings may be continuous with the distal end or positioned separately along the cannula wall. Their longitudinal edges are sharpened to function as cutting blades. During forward motion, tissue enters through both the distal and side openings. Rotating the cannula around the insertion axis causes the side- opening edges to sever tissue circumferentially.

A variation described by Mangat et al. (34) involves a biopsy needle for liver sampling via blood vessels. This design features both an inner and outer cannula, each with a side opening continuous with the distal end and spanning approximately half the circumference (see Figure 4G). During forward motion, the cannulas are aligned to form a half-open tip. The outer cannula is then rotated around the insertion axis, producing circumferential cutting and a scissor-like action against the inner cannula.

Another common design for forward and circumferential cutting employs screw-like mechanisms. These designs include cutting members shaped as an open helix (79), an open helix terminating in a solid piercing tip (60), or a structure resembling an Archimedean screw (54). In all cases, the outer circumferential edges of the helix are sharpened to act as blades. Sampling involves advancing the needle through simultaneous rotation and forward motion, effectively screwing it into the tissue. It then continues rotating in place to fully sever any remaining connections to the surrounding tissue. Depending on the design, this mechanism yields either a cylindrical core sample or a continuous helical strand of tissue. Some patents describe screw-like structures used solely for anchoring the needle in tissue, with a separate mechanism responsible for cutting. These designs are discussed under gripping mechanisms in Section 4.

Two patents stand out as exceptions within the group of forward and circumferential cutting. While their designs appear quite different at first glance, both are based on a shared concept: a rigid hollow cannula incorporating partial U-shaped cut-outs along its wall, which form compliant cutting segments (see Figure 4H). These segments are designed to flex either radially inward (81) or radially outward (66) and sever tissue circumferentially when the cannula is rotated around its insertion axis.

Most circumferential cutting designs rely on full rotary motion at the needle tip, which is typically actuated from the proximal end of the device outside the patient's body. Transmitting this motion through a fully flexible shaft presents considerable design challenges, particularly due to the shaft's limited torsional stiffness (84, 85). These actuation constraints will be described in more detail in Section 5.3.1.1.

#### 3.2.3 Forward, radial & circumferential cutting

While the previous sections described needle designs that combine forward motion with either radial or circumferential cutting, one patent (64) explicitly integrates both strategies within a single device (see Figure 4I). This design does not introduce a fundamentally new approach beyond those outlined in Sections 3.2.1 and 3.2.2. Instead, it combines compliant cutting segments that bend radially inward with longitudinal slits that enable circumferential tissue severance when the needle tip is rotated around its insertion axis.

## 4 Gripping the biopsy sample

In endoscopic or catheter-based biopsy procedures, achieving sufficient grip on the sample is critical at multiple stages. Before and during cutting, grip stabilizes the target tissue by counteracting tissue displacement. After cutting, it secures the sample within the needle during retraction. Adequate grip can also aid in shearing any residual connections between the sample and surrounding tissue. Having established the importance of tissue grip, the following sections describe the mechanisms by which the needle can apply grip to the biopsy sample. Representative examples of each gripping strategy are presented in Figure 5.

### 4.1 Gripping from the back

#### 4.1.1 Suction-based gripping

Seventeen patents (20–22, 31, 38–40, 46, 49, 50, 54, 61, 62, 64, 72, 77, 83) describe the use of suction to apply grip on the tissue sample from the back. Suction is often used in combination with other gripping strategies. It is typically generated within the lumen of the biopsy needle by applying negative pressure, for example by attaching a syringe to the proximal end of the device located outside the patient's body (see Figure 5A). Negative pressure can be transmitted through air, referred to as dry-suction, or through fluid, referred to as wet-suction (11). In wet-suction, the needle is first flushed with a solution such as saline before cutting the target tissue. This allows the negative pressure to act on the incompressible fluid.

Suction may be applied at different stages of the sampling process. It can be used before cutting to draw tissue into the lumen, during cutting to counteract tissue displacement, and after cutting to retain the sample within the needle. The use of suction has been shown to improve sample adequacy, with wet-suction providing superior histological integrity compared to dry-suction (11).

### 4.2 Gripping from the front

#### 4.2.1 Macroshape gripping

Twenty-eight patents (15, 17–19, 24, 25, 30, 31, 34, 35, 37, 44, 47, 51, 59, 60, 63, 64, 66, 68–71, 73–76, 81) describe the use of a macroshape grip applied from the front of the biopsy sample, where “front” refers to the distal end of the needle. In these designs, the sample is partially or fully enclosed from the front by large structural elements that hook around it.

The most common approach (15, 17–19, 24, 25, 30, 34, 35, 44, 47, 51, 59, 60, 63, 66, 74, 76) involves an inner needle terminating in a solid, harpoon-like tip. This tip may be centrally connected to an actuating rod (see Figure 5B) or integrated into a solid stylet featuring a large lateral recess. In both cases, the inner needle is housed within an outer cannula. During sampling, the tip is advanced into the tissue at high speed, often using a spring-based mechanism. This action positions the target tissue between the tip and the outer cannula. Cutting is then achieved either by retracting the tip (backward cutting) or by advancing the outer cannula (forward cutting). In both cases, the sample is fully enclosed from the front by the harpoon-like tip and secured at the side within the outer cannula.

In these designs, macroshape gripping plays a role both during and after cutting. During cutting, the tissue is held between the tip and the cannula, which creates a counterforce that helps prevent tissue displacement. After cutting, the sample remains securely held within the needle tip. A well-known commercial implementation of this gripping strategy is the tru-cut-style needle tip, which is used in catheter-based procedures such as transvenous liver biopsy (8).

An alternative approach to achieving macroshape grip from the front involves biopsy needles with inward-bending cutting segments (see Figures 3D,F), as described in Section 3.2 (37, 64, 66, 68–71, 73, 75, 81). These segments serve a dual purpose: they first cut the sample through radial or circumferential motion, and subsequently partially enclose it from the front. In these designs, macroshape grip functions only after cutting, primarily during needle retraction, where it helps secure the sample within the lumen and shear any residual tissue connections. One patent (31) describes a structurally similar macroshape grip mechanism, in which compliant inward-bending segments in the needle wall enclose the sample from the front but do not contribute to cutting. In this case, the structures are solely intended to grip the sample.

### 4.3 Gripping from within

#### 4.3.1 Microshape gripping

Four patents (23, 43, 71, 72) describe needle designs that achieve grip from within the sample using microshape mechanisms. These designs employ small features that superficially interlock with the tissue. As discussed in Section 2.4, the distinction between microshape and macroshape grip is not always clear-cut. We classified designs to the best of our judgment based on the relative feature size depicted in the patent drawings.

Microshape grip designs typically incorporate a small anchoring element inserted into the target tissue, mechanically interlocking with the sample prior to cutting. The cutting element is then advanced over the anchor, severing the sample either through forward motion alone or in combination with radial or circumferential cutting. The anchoring element stabilizes the tissue during cutting, secures the sample within the needle, and assists in shearing residual tissue connections during retraction. However, because the anchor is positioned centrally within the sample, it may yield a less intact tissue core, compromising histological integrity. This is discussed in more detail in Section 5.3.3.

Three patents describe anchoring elements composed of a stylet or wire equipped with microbarbs (23, 43) (see Figure 5C) or radial protrusions (71), which enter the tissue via a simple forward motion. Two patents (43, 72) detail mini screw-threaded or helical elements that require rotational advancement to achieve anchoring. As previously noted, generating full rotary motion at the tip of a flexible instrument presents significant actuation challenges.

#### 4.3.2 Macroshape gripping

Five patents (27–29, 54, 72) describe macroshape grip mechanisms applied from within the sample, all featuring screw-like designs housed inside an outer cutting cannula (see Figure 5D). Similar to the mini screw-threaded microshape grips discussed in Section 4.3.1, these designs require rotary motion to anchor the needle prior to cutting. Some of these screw-like configurations are also discussed in Section 3.2.2 on forward and circumferential cutting, while others function solely as a grip mechanism and must be paired with a separate cutting mechanism to separate the sample from surrounding tissue.

Although screw-like designs offer substantial grip, they might compromise sample quality. In these mechanisms, the gripping element is fully screwed through the center of the sample prior to cutting, which hinders the retrieval of an intact, cylindrical core. Additionally, these designs inherit the previously noted challenges associated with generating rotary motion at the tip of a flexible shaft.

### 4.4 Gripping from the side

#### 4.4.1 Friction-based gripping

Fifteen patents (16, 26, 33, 34, 37, 41, 42, 52, 60, 67, 71, 72, 78, 80, 82) describe gripping mechanisms that rely on friction between the sample and the needle wall. This type of grip can only be applied once the sample is at least partially positioned within the needle lumen. As a result, it cannot prevent tissue displacement out of the needle during the initial cutting motion but does assist in retaining the sample during needle retraction. It should be noted that all biopsy needles discussed in this review inherently involve some degree of friction-based grip, as friction is always present between contacting surfaces. However, the patents classified under this group either explicitly aim to increase friction or rely solely on friction-based grip due to the absence of other gripping mechanisms.

Six patents (26, 37, 71, 72, 78, 80) actively enhance surface friction by increasing the normal force on the sample through compression. In most designs, this is achieved using compliant cutting segments that expand outward during cutting and are subsequently compressed inward by an outer cannula, thereby reducing the lumen diameter (see Figure 5E). These cutting mechanisms are described earlier in Section 3.2. In contrast, one patent (26) presents a needle which is shaped as a rigid hollow cannula with an open distal end and a lumen that narrows proximally, compressing the sample as it is advanced further into the needle.

The remaining nine patents (16, 33, 34, 41, 42, 52, 60, 67, 82) were classified as relying on friction-based grip, as they feature open-ended hollow cannulas without any additional retention-enhancing mechanisms. Consequently, these designs depend solely on surface friction between the sample and the inner wall of the needle to maintain grip.

#### 4.4.2 Microshape gripping

Eight patents (26, 30, 37, 38, 45, 48, 53, 71) describe the use of microshape grip to secure the sample from the side. Similar to friction-based grip, this mechanism engages as soon as the tissue contacts the inner surface of the needle. As previously discussed, the boundary between microshape and macroshape grip is not sharply defined. We classified designs to the best of our judgment based on the relative feature size depicted in the patent drawings. Microshape grip mechanisms typically feature very small elements, such as microbarbs or scales (see Figure 5F), positioned proximally on the inner surface of the needle tip (26, 30, 37, 38, 48, 71). These microbarbs are typically formed by creating partial U-shaped cut-outs in the needle wall and bending them slightly inward. Most patents do not specify the elasticity of these microfeatures, although this property significantly affects their function.

In designs with flexible microstructures, the proximally oriented features flatten against the needle wall during insertion, allowing smooth entry of the tissue. During retraction, the features partially lift and mechanically interlock with the sample, creating direction-dependent resistance: movement is easy in the forward (insertion) direction but resisted in the backward (retraction) direction. This enhances sample retention within the needle. In contrast, rigid microstructures do not flatten during insertion, resulting in greater resistance as the tissue enters the needle. Although retention is still achieved through mechanical interlocking, the direction-dependent effect is less pronounced.

Two patents (45, 53) describe an alternative approach that does not involve protruding features. Instead, they introduce indentations or a small hole in the needle wall (see Figure 5G). In these designs, the sample slightly bulges into the recessed area, mechanically interlocking with borders of the hole in the needle wall and providing microshape grip from the side of the sample.

#### 4.4.3 Macroshape gripping

Seven patents (25, 28, 32, 36, 60, 65, 79) describe designs that apply macroshape grip from the side of the sample. The most common approach involves a centrally open helical element that is advanced into the tissue using rotary motion, similar to a corkscrew (28, 32, 36, 60, 79). This helical structure may serve as part of the cutting mechanism, enabling forward and circumferential cutting, or it may function solely as a gripping element, with a separate cutting component used to sever the sample from surrounding tissue. The sample is retained within the open core of the helix, with tissue bulging radially outward between the spiral turns. This configuration creates a large mechanically interlocking surface along the side of the sample, contributing to grip before, during, and after cutting.

In contrast, Roxhed et al. (65) present an interesting alternative macroshape gripping strategy, involving a needle tip with multiple compliant cutting segments that expand radially outward when extended beyond a constraining outer cannula (see Figure 5H). These segments feature large proximally oriented inner barbs. During forward advancement, the barbs slide relatively easily through the tissue as the segments expand. Upon retraction, the outer cannula forces the segments to bend radially inward, causing the barbs to mechanically interlock with the sample and shear any remaining tissue connections. A similar mechanism is described in one other patent (25).

## 5 Discussion

### 5.1 Main findings

This study provides an overview of patent literature on flexible biopsy needles for endoscopic and catheter-based procedures, focusing on biopsy mechanisms at the needle tip. Using a classification-based search in Espacenet, we identified 458 patents, of which 65 met the inclusion criteria. We classified these patents according to the design strategies employed for cutting and gripping the biopsy sample. Cutting mechanisms were classified as either unidirectional, such as forward or backward cutting, or multidirectional, combining forward cutting with radial or circumferential motion. Gripping strategies were categorized based on the location of grip application: from the back, front, within, or side of the sample. Forward-cutting needles combined with suction or macroshape grip mechanisms that engage the sample from the front dominate the patent landscape, likely due to their mechanical simplicity. However, our analysis also reveals a wide variety of alternative cutting and gripping approaches that offer promising directions for future design.

### 5.2 Comparative analysis

This study reveals a field predominantly driven by industry, with 78% of filings originating from companies, as compared to 12% from individual applicants and only 10% from academic institutions. Geographically, patent activity is largely concentrated in a small number of countries, with the United States accounting for 43% of filings, followed by Japan with 26%. The remaining patents are distributed across a small number of countries in Europe and Asia. No patents were identified from Africa, Oceania or South America.

In terms of application, the majority of patents (83%) focuses on endoscopic procedures, such as EUS and EBUS-guided biopsy, which use the GI tract and the airways as pathways for navigation. In contrast, catheter-based approaches, which navigate through blood vessels, account for just 17% of the reviewed patents. This distribution indicates that developmental efforts in flexible biopsy needle technology are primarily focused on endoscopic applications.

**Figure 6:**
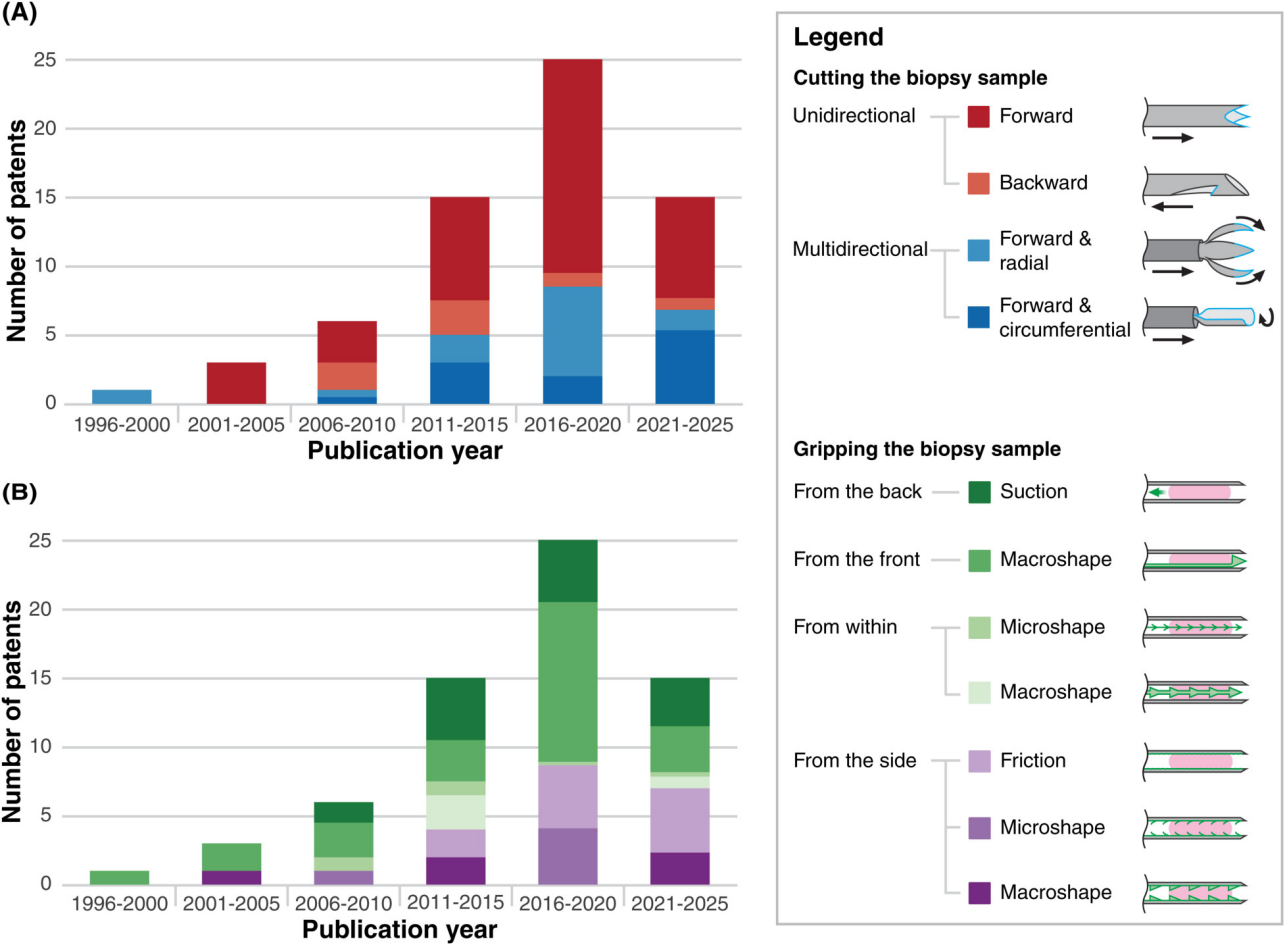
Temporal distribution of the included patents, categorized by **(A)** cutting and **(B)** gripping methods. Patents describing multiple designs or combined approaches are counted once within each classification framework (cutting or gripping), with fractional values assigned when multiple methods are reported (e.g., a single patent may contribute ½ forward and ½ backward in the cutting framework, and 1 to suction in the gripping framework).

Historically, however, the origins of this technology lie in catheter-based biopsy. The earliest known flexible biopsy needle was developed by Charles Dotter in 1964 for transvenous liver biopsy in dogs (86), marking a significant academic contribution. In 1978, Gilmore et al. (87) advanced this concept by integrating a tru-cut-style biopsy tip on the shaft of flexible endoscopy forceps. A pivotal moment occurred in 2004 when Cook Medical (Bloomington, Indiana, USA) patented and released the Quick-Core® biopsy needle (17). This device adapted the tru-cut principle for endoscopic and catheter-based procedures and is classified in this review as a forward-cutting needle with macroshape grip applied from the front. Its introduction marked a transition from academic prototypes to clinically viable products and initiated a surge in patent activity that peaked between 2016 and 2020 (Figure 6).

This growth period coincides with the clinical adoption of EUS-guided FNB, which enabled core tissue sampling and gradually replaced EUS-guided FNA, limited to cell or fluid sampling (11). Patents from this time predominantly feature forward-cutting designs combined with macroshape grip mechanisms that engage the sample from the front, often resembling tru-cut needle configurations. Other designs pair forward-cutting mechanisms with suction or friction-based gripping. This approach remains commercially dominant in current FNB needle designs (11), with many optimizing the geometry of the cutting edges to reduce insertion force, as discussed in Section 3.1.1.

Between 2006 and 2015, backward-cutting needle designs briefly emerged in the patent landscape. However, their prevalence declined after 2016, likely due to the limited clinical impact of the backward-cutting ProCore® needle (Cook Medical), which failed to demonstrate superior diagnostic accuracy over EUS-FNA (11).

The decline in patent filings from 2021 onward is difficult to interpret. One possible explanation relates to the publication timeline of international patent applications filed under the WIPO. Such applications typically take 18 months before they are published and become accessible in databases like Espacenet (88). As a result, there is an inherent delay between the filing date (priority date) and the publication date, which may mean that relevant inventions falling within the scope of this review have not yet been publicly disclosed at the time of writing. Beyond this publication delay, the observed decline may also reflect broader trends in the field. It could indicate market saturation or a stabilization following the rapid expansion of EUS-FNB technologies. Alternatively, emerging clinical interest in transvenous biopsy for abdominal and pelvic lymph node sampling (9, 10) could signal a renewed focus on catheter-based approaches, similar to how the first academic attempts by Charles Dotter initiated the development of this field (86).

Patent trends also reveal a clear preference for mechanically simple designs. Simple designs generally require only a limited number of geometric features to define all parts, which correlates with fewer total components and fewer interactions between them (89). In this review, the type of movement required at the needle tip is also considered a contributor to mechanical simplicity. In line with this, forward-cutting needles (56%), macroshape grip from the front (36%), and suction from the back (22%) dominate both the patent landscape and the commercial market. These devices typically consist of a hollow cutting cannula with an open distal end, and their gripping mechanisms are actuated through straightforward means, such as a push-pull motion or syringe-based suction. Their mechanical simplicity supports ease of manufacturing, an aspect that will be further discussed in Section 5.3.2.

At the same time, the patent landscape also reveals a reservoir of untapped innovation. Multidirectional cutting needles, often incorporating compliant structures that expand radially or circumferentially, aim to increase tissue yield without enlarging the insertion profile. Designs with multiple grip mechanisms, including those acting from within or from the side of the sample, propose alternative strategies for improving sample retention and potentially enhancing diagnostic accuracy. These mechanisms represent a broader conceptual space for future development. Their potential for clinical translation, along with the practical considerations that influence which innovations are likely to progress towards commercial products, will be examined in the next section.

### 5.3 Clinical translation

Patent applications generally do not include experimental validation of technical or clinical performance. Instead, they primarily communicate the conceptual design ideas that applicants seek to protect for potential future commercialization. As such, the patent data analyzed in this review do not allow us to directly assess how feasible clinical translation of specific design strategies will be. Any conclusions about translation potential must therefore remain partly interpretive.

Nevertheless, several engineering and clinical factors strongly influence whether a conceptual mechanism is likely to perform reliably across different clinical procedures. In this section, we focus on three such factors: (1) constraints imposed by flexible shafts, (2) manufacturability & mechanical feasibility, and (3) biopsy sample quality. We discuss how these factors shape the relative translation potential of the design strategies found in the analyzed patent applications.

#### 5.3.1 Constraints imposed by flexible shafts

##### 5.3.1.1 Constraints due to torsion

The feasibility of needle tip design strategies must be evaluated in relation to the constraints imposed by the flexible needle shaft. In FNB and TVB needles the biopsy tip is relatively rigid, while the hollow shaft remains flexible to enable navigation through tortuous anatomy. It is therefore important to consider these constraints in the context of actuation, specifically how motion generated at the handle is transmitted through a long, thin, flexible shaft. In endoscopic and catheter-based procedures, the long flexible needle shaft with a very small diameter exhibits low torsional stiffness, which means that rotation applied at the handle does not reliably translate into well-controlled rotational motion at the needle tip. In contrast, a push–pull motion applied at the handle is transmitted more reliably as axial translation along the shaft. This motion can be transmitted by an axially stiff cable inside the needle shaft that moves with respect to the shaft and delivers translational motion to the mechanism at the tip. As a result, tip mechanisms that rely on axial translation for their cutting or gripping motions are generally more compatible with flexible biopsy needles.

For all unidirectional cutting designs, motion is directly driven by axial translation. For multidirectional cutting designs, feasibility depends on how the cutting motion at the tip is generated. In the forward and radial cutting strategies shown in Figures 4A–D,E, the radial cutting motion is initiated by axial translation, and therefore these concepts are not limited by poor torsional transmission. In contrast, the forward and radial cutting design in Figure 4C requires distal rotary motion to initiate its radial cutting trajectory, making it less suited for translation into a flexible biopsy needle for endoscopic or catheter-based procedures.

A similar distinction applies to forward and circumferential cutting. Most circumferential designs require a full rotary motion at the tip and are therefore poorly compatible with flexible biopsy needle procedures. The design shown in Figure 4F, however, is a notable exception: circumferential closure of a compliant inner needle is driven by pure axial translation of an outer cannula, making this concept compatible with flexible shafts despite their low torsional stiffness.

These actuation-related limitations also affect gripping strategies. Mechanisms that require controlled distal rotation, such as screw-based anchors intended to apply micro- or macroshape grip from within the sample (Figure 5D), or helical macroshape grips applied from the side, are similarly constrained by the inability to transmit reliable torque through a flexible shaft.

##### 5.3.1.2 Constraints due to buckling

Beyond the challenge of transmitting controlled motion through a flexible shaft, it is also important to consider how the mechanical support provided by the introducer influences needle stability during tissue interaction. Because introducers vary widely in stiffness, the stability they offer to the flexible needle shaft differs substantially between catheter-based and endoscopic procedures. These differences in support conditions influence which cutting and gripping strategies are most feasible in each procedure.

In catheter-based procedures, the needle shaft is only minimally supported by the thin, highly flexible vascular introducer sheath. Because both the catheter and vessel wall are compliant, the needle experiences very limited lateral support, and the effective unsupported length of the shaft remains relatively long. Under these conditions, when axial force is applied to initiate cutting, the flexible needle may buckle rather than advance. In this setting, gripping mechanisms that anchor in the tissue before cutting can be advantageous, as they establish a local counterforce at the tip. This counterforce stabilizes the distal end of the needle, reduces the risk of buckling, and improves cutting efficiency. Examples include gripping mechanisms that apply grip from the front of the sample (Figure 4B) or mechanisms that apply grip from within the sample, such as the internal anchor shown in Figure 5C. Such designs may therefore have particularly high relevance in catheter-based biopsy procedures where the shaft lacks external support.

In EBUS/EUS procedures, the mechanical environment differs substantially. Here, the needle shaft is supported along most of its length by the relatively thick and stiff endoscope. The rigidity of the endoscope provides strong lateral constraint and significantly shortens the effective unsupported length of the needle. As a result, the likelihood of shaft buckling when encountering tissue resistance is reduced. In this more stable, mechanically supported environment, gripping mechanisms that penetrate the tissue before cutting become less essential because the introducer itself provides sufficient stability for controlled tissue penetration and cutting.

#### 5.3.2 Manufacturability & mechanical feasibility

The ease of manufacturing a design strongly influences the likelihood that a device can be commercialized. Designs that are simpler to manufacture generally result in lower production costs and therefore offer greater commercial viability (90). Flexible biopsy needles are generally intended for single use (91), which makes low production cost an essential requirement. Because most patent applications do not include manufacturing details, we focus here on general manufacturability principles and our own design expertise at the small scale at which these biopsy mechanisms typically operate, approximately 1 mm in diameter (8, 11).

Designs that consist of multiple interacting parts with tight tolerances, or parts with complex spatial geometries, often have a reduced translation potential due to manufacturing constraints. At these small dimensions, reduced tolerance margins require high-precision machining, which increases production costs (90). Additionally, components that require multistep micro assembly significantly increase assembly time and thus costs per unit. An example is the design shown in Figure 4D, which incorporates a small, separate cutting element mounted inside a hollow cannula and rotating around an internal linking pin. This mechanism would require multistep micro assembly and extremely precise alignment, reducing its translational potential.

By contrast, designs that use thin walled tubular structures as their primary architecture tend to be easier to manufacture at this scale. Thin-walled tubes can be produced through conventional tube-drawing processes, after which features can be added using laser micromachining, electrical discharge machining (EDM), or computer numerical control grinding to achieve high-precision, beveled cutting edges (92, 93). Examples of cutting strategies that are compatible with this approach include those shown in Figures 3A,B, Figures 4A,G,I. Among gripping strategies, this also applies to microshape grip features formed by a small hole in the needle wall, such as in Figure 5G.

However, not all tube-based mechanisms are straightforward to manufacture. Internal structures located inside the needle lumen, such as the microshape gripping scales in Figure 5F, must be fabricated on the inner surface of a tiny hollow tube. Creating such features is technically extremely challenging, if not practically impossible, using current micromanufacturing techniques. These limitations substantially reduce the translation potential of designs that rely on complex internal structuring within confined geometries.

Moreover, design strategies that use compliant cutting segments face inherent mechanical trade-offs. Effective cutting requires sufficient stiffness, whereas bending requires flexibility. When a mechanism depends on large bending deformation, its structural stiffness is reduced, which limits its ability to cut tissue efficiently. As a result, some designs, such as the highly deformable compliant cutter in Figure 4F, may struggle to maintain enough stiffness to penetrate tissue while also performing a large circumferential bending motion. In contrast, compliant mechanisms used in certain forward and radial cutting designs, such as the example in Figure 4A, require only moderate bending deformation. This allows a more feasible balance between flexibility for the bending motion and stiffness for effective cutting.

#### 5.3.3 Biopsy sample quality

Biopsy sample quality depends on how effectively the device cuts and on how well it minimizes mechanical stress that can damage tissue. Two common forms of damage that reduce histological quality are fragmentation and compression artifacts. Fragmentation occurs when the tissue breaks into separate pieces (94), while compression artifacts occur when tissue is locally squeezed, which smudges the outer cell layers (95). Because patents do not report diagnostic outcomes, the influence of specific design features on sample quality cannot be directly assessed. Even so, general mechanical principles allow us to reason how certain design features may affect the risk of fragmentation and compression artifacts.

To understand how design choices influence damage, it is first necessary to consider the mechanical forces that can fragment tissue. Based on fundamental tissue biomechanics, tensile, shear, compressive and torsional stresses applied to the sample can all contribute to fragmentation (96). A logical implication is that designs that limit these forces during cutting and gripping may help reduce fragmentation.

One strategy to reduce these forces is to lower the cutting resistance of the needle. For example, optimizing the sharpness and geometry of beveled cutting edges has been shown to decrease cutting force (93). Lower cutting forces reduce the deformation imposed on the tissue during initial cutting, which may limit fragmentation. Although this principle applies to cutting edges in any direction of motion, such optimization was only found in the forward cutting designs included in this review (see Section 3.1.1).

Cutting forces are only one contributor to sample fragmentation, because the way a device grips and stabilizes tissue can also introduce stresses. Designs that minimize the moving contact surface relative to the sample likely reduce frictional forces and therefore lower stress. In contrast, internal anchors or screw-based gripping mechanisms increase the contact surface to generate local counterforces that may stabilize the sample for cutting (Figures 5C,D), but they also introduce additional internal stresses. The combined effects of penetration, rotation and withdrawal may therefore increase the risk of fragmentation. Compression-based gripping mechanisms, such as the example shown in Figure 5E, may further contribute to compression artifacts if the applied compressive forces are high.

The impact of these cutting and gripping mechanisms depends strongly on the mechanical properties of the target tissue, which complicates general predictions about sample quality. High grade tumors in certain cancers can be more friable (97), and fibrotic tissue fragments more readily (94, 98). A clinical illustration of this tissue dependence is found in liver biopsy. In non-fibrotic liver tissue, suction applied from the back of the sample increases tissue yield, but fragmentation becomes more likely with higher degrees of liver fibrosis (94). A plausible explanation is that suction introduces tensile forces that fibrotic tissue cannot withstand. This indicates that suction-based gripping strategies are not universally applicable and should be adapted to the mechanical properties of the target tissue. Taken together, these observations show that the interaction between device design and tissue type is critical, because similar cutting or gripping mechanisms can influence sample quality very differently across tissues.

#### 5.3.4 Clinical translation conclusion

Although the patent data remain partly interpretive, our analysis highlights a set of general features that make some patented designs more suited for clinical translation than others. The low torsional stiffness of flexible shafts limits precise torque transmission and makes axial translation the most dependable form of actuation. Cutting forces should be minimized to limit tissue deformation, and gripping strategies should avoid excessive frictional or compressive loading. In catheter-based procedures the long unsupported shaft increases the risk of buckling, which makes the addition of stabilizing anchors a useful gripping strategy, although they can also introduce internal stresses that increase the likelihood of fragmentation. Manufacturability at the millimeter scale favors simple tubular architectures with 2D wall features added by laser micromachining or EDM, whereas designs that require intricate micro assembly or complex internal structures have low feasibility. Compliant mechanisms also require careful evaluation because large bending deformations reduce structural stiffness and limit cutting efficiency, while designs that rely on moderate bending can achieve a workable balance between flexibility and cutting performance. Ultimately, biopsy tips are not a one size fits all solution, and their effectiveness depends strongly on the clinical procedure and the mechanical properties of the target tissue.

### 5.4 Limitations

This review relied on international patent applications filed under WIPO and restricted the analysis to patents available in English. Although this strategy captures key technological developments, since these inventions undergo extensive assessment by international searching authorities to determine patentability, it may have introduced a geographic selection bias. According to the WIPO statistics database, in 2024 the regions Oceania, South America and Africa together accounted for only 1.3% of all international patent applications (99). Innovators in these regions may more often file their patents only with national or regional offices rather than through the international route. As a consequence, innovations originating from these regions may be underrepresented in this study.

Furthermore, patents focused solely on fluid aspiration for cell sampling were excluded. Although these devices primarily use suction as a gripping strategy, they may include valuable cutting features that were not captured in this review. Additionally, this study focused on mechanisms at the needle tip, omitting improvements to the shaft or proximal end, which could significantly affect overall performance but fall outside the scope.

Finally, although many patents propose novel mechanisms for cutting and gripping biopsy samples, their clinical utility remains uncertain due to the lack of experimental validation and performance data, a limitation inherent to patent literature. It is therefore important to note that patent frequency does not necessarily reflect clinical effectiveness or clinical adoption, but rather represents innovation activity and the legal protection of ideas.

### 5.5 Future outlook

This review highlights a gap between patented innovations and commercially available flexible biopsy needles. While many patents introduce novel cutting and gripping mechanisms, their clinical utility remains uncertain due to limited validation. Nonetheless, strong industry involvement suggests that some innovations may reach clinical practice, although development and regulatory approval remain costly and time-intensive (100).

To date, commercial innovation has largely focused on incremental improvements, such as refining the cutting edges of end-cutting FNB needles to reduce insertion force. In contrast, the patent landscape reveals more radical strategies. These include the integration of compliant structures that allow the needle tip to expand radially or circumferentially during cutting, thereby increasing tissue yield without enlarging the insertion profile. Such designs also apply slight compression to the sample after cutting, which enhances friction between the needle and tissue and may improve sample retention. Together, these mechanisms represent a conceptual shift from rigid to compliant biopsy needle designs.

Compliant materials and deployable structures are already well-established in other catheter-based interventions, such as stent deployment (101) and thrombus retrieval (102), where devices are introduced in a compact form and expand at the target site. Applying these principles to biopsy needle design could result in devices that are both mechanically simple and functionally advanced. However, challenges related to manufacturing complexity and material durability must be addressed before clinical translation is feasible.

Parallel developments in navigation and real-time imaging may further support the advancement of flexible biopsy needle technology. Although EUS and EBUS provide high-resolution imaging, the relatively large diameter of the endoscope restricts access to narrow lumens and deep-seated lesions. Advances in miniaturized steerable introducers, such as steerable catheters (103–105), could extend the reach of minimally invasive procedures and enable sampling in anatomically complex regions.

Real-time imaging modalities like magnetic resonance imaging (MRI) also hold promise for improving targeting accuracy in deep-seated lesions, owing to MRI's superior soft-tissue contrast compared to ultrasound or computed tomography (106). However, MRI-guided biopsy would require the development of MRI-compatible flexible needles, introducing additional engineering and material challenges. Furthermore, MRI is considerably more expensive than ultrasound (107), and its clinical use may only be justified in cases where other imaging modalities are insufficient.

Taken together, these developments point toward an innovation pathway that combines optimization of cutting edge geometry, radially expanding compliant tip designs, advanced navigation tools and real time imaging modalities. Continued refinement of these elements over the coming years may enable more precise and effective biopsies, particularly for anatomically challenging or otherwise inaccessible lesions.

## 6 Conclusion

This review of 65 patents provides a structured overview of flexible biopsy needle designs for endoscopic and catheter-based procedures, focusing on biopsy mechanisms at the needle tip. Forward-cutting needles combined with suction or macroshape grip mechanisms that engage the sample from the front dominate current designs, but numerous alternative approaches offer promising design opportunities. By highlighting the diversity of technical solutions, this review may guide the development of next-generation flexible biopsy needles for minimally invasive cancer diagnosis, particularly in anatomically challenging regions. Realizing this potential will require close collaboration between engineers and clinicians to bridge the gap between innovative design and clinical application.
