# Supplementary material for: Design strategies for flexible core biopsy needles: insights from patent literature
Source: Front Med Technol. 2026 Apr 1;8:1771655. doi: 10.3389/fmedt.2026.1771655 (PMC13079630; doi:10.3389/fmedt.2026.1771655)
Supplement: Supplementary file 1 [file Table1.pdf]

Supplementary Material

Supplementary Table 1. Overview of all patents included in this review, detailing their publication year, country of origin, assignee type, and design strategies for cutting and gripping biopsy samples.

Patent	Year	Origin	Assignee	Cutting Strategies	Gripping Strategies
WO9835615A1 (75)	1998	USA	Individual Applicant	Multidirectional - Forward & radial	From the front - Macroshape
WO02065919A1 (36)	2002	Belgium	Individual Applicant	Unidirectional - Forward	From the side - Macroshape
WO02060312A2 (18)	2002	USA	Industry	Unidirectional - Forward	From the front - Macroshape
WO2004062505A1 (17)	2004	USA	Industry	Unidirectional - Forward	From the front - Macroshape
WO2007010796A1 (20)	2007	Japan	Industry	Unidirectional - Forward	From the back - Suction
WO2007014313A2 (63)	2007	USA	Industry	Unidirectional - Backward	From the front - Macroshape
WO2007084910A2 (24)	2007	USA	Industry	Unidirectional - Forward<br>Unidirectional - Backward	From the front - Macroshape
WO2009151883A1 (64)	2009	USA	Industry	Multidirectional - Forward & radial<br>Multidirectional - Forward & circumferential	From the front - Macroshape<br>From the back - Suction
WO2010144278A1 (23)	2010	USA	Industry	Unidirectional - Forward	From within - Microshape
WO2010113080A2 (45)	2010	Israel	Individual Applicant	Unidirectional - Forward<br>Unidirectional - Backward	From the side - Microshape

WO2011053648A1 (43)	2011	USA	Industry	Unidirectional - Forward	From within - Microshape
WO2011126109A1 (22)	2011	Japan	Industry<br><br>Academic Institution	Unidirectional - Forward	From the back - Suction
WO2011126963A2 (62)	2011	USA	Industry	Unidirectional - Backward	From the back - Suction
WO2012133276A1 (49)	2012	Japan	Industry	Unidirectional - Forward<br>Unidirectional - Backward	From the back - Suction
WO2012014773A1 (77)	2012	Japan	Industry	Multidirectional - Forward & circumferential	From the back - Suction
WO2013168166A1 (25)	2013	Israel	Industry	Unidirectional - Forward	From the front - Macroshape<br>From the side - Macroshape
WO2014027548A1 (76)	2014	Japan	Industry	Multidirectional - Forward & radial	From the front - Macroshape
WO2014054525A1 (59)	2014	Japan	Industry	Unidirectional - Backward	From the front - Macroshape
WO2014080366A1 (29)	2014	Netherlands	Industry	Unidirectional - Forward	From within - Macroshape
WO2014192646A1 (78)	2014	Japan	Industry	Multidirectional - Forward & circumferential	From the side - Friction
WO2014194146A2 (31)	2014	USA	Industry	Unidirectional - Forward	From the front - Macroshape<br>From the back - Suction
WO2015189067A1 (27)	2015	Sweden	Individual Applicant	Unidirectional - Forward	From within - Macroshape
WO2015076936A1 (79)	2015	USA	Industry	Multidirectional - Forward & circumferential	From the side - Macroshape
WO2015073675A1 (28)	2015	USA	Industry	Unidirectional - Forward	From within - Macroshape<br>From the side - Macroshape

WO2016025166A1 (70)	2016	USA	Industry	Multidirectional - Forward & radial	From the front - Macroshape
WO2016027854A1 (53)	2016	Japan	Industry	Unidirectional - Forward	From the side - Microshape
WO2014007380A1 (67)	2014	Japan	Academic Institution	Multidirectional - Forward & radial	From the side - Friction
WO2016019097A1 (74)	2016	USA	Industry	Multidirectional - Forward & radial	From the front - Macroshape
WO2016010735A1 (71)	2016	USA	Industry	Multidirectional - Forward & radial<br>Multidirectional - Forward & circumferential	From the front - Macroshape<br>From within - Microshape<br>From the side - Friction<br>From the side - Microshape
WO2016048898A2 (30)	2016	USA	Industry	Unidirectional - Forward<br>Multidirectional - Forward & circumferential	From the side - Microshape
WO2016044600A2 (38)	2016	USA	Industry	Unidirectional - Forward	From the back - Suction<br>From the side - Microshape
WO2016199597A1 (35)	2016	Japan	Industry	Unidirectional - Forward	From the front - Macroshape
WO2016196913A1 (48)	2016	USA	Industry	Unidirectional - Forward	From the side - Microshape
WO2016154170A1 (37)	2016	USA	Industry	Unidirectional - Forward<br>Multidirectional - Forward & radial	From the front - Macroshape<br>From the side - Friction<br>From the side - Microshape
WO2016132577A1 (40)	2016	Japan	Industry	Unidirectional - Forward	From the back - Suction
WO2016144834A1 (68)	2016	USA	Industry	Multidirectional - Forward & radial	From the front - Macroshape
WO2018115368A1 (19)	2018	Ireland	Academic Institution	Unidirectional - Forward	From the front - Macroshape
WO2018148431A1 (44)	2018	USA	Academic Institution	Unidirectional - Forward	From the front - Macroshape

WO2018151943A1 (50)	2018	USA	Industry	Unidirectional - Forward	From the back - Suction
WO2018203138A1 (69)	2018	Japan	Industry	Multidirectional - Forward & radial	From the front - Macroshape
WO2018098271A1 (66)	2018	USA	Industry	Multidirectional - Forward & radial<br>Multidirectional - Forward & circumferential	From the front - Macroshape
WO2019157389A1 (33)	2019	Japan	Industry	Unidirectional - Forward	From the side - Friction
WO2019155472A1 (26)	2019	Israel	Industry	Unidirectional - Forward	From the side - Friction<br>From the side - Microshape
WO2019103694A1 (34)	2019	Singapore	Industry<br><br>Academic Institution	Unidirectional - Forward<br>Multidirectional - Forward & circumferential	From the front - Macroshape<br>From the side - Friction
WO2020003198A1 (47)	2020	Italy	Industry	Unidirectional - Forward	From the front - Macroshape
WO2020019308A1 (73)	2020	China	Individual Applicant	Multidirectional - Forward & radial	From the front - Macroshape
WO2020089422A1 (52)	2020	Sweden	Individual Applicant	Unidirectional - Forward	From the side - Friction
WO2020234919A1 (21)	2020	Japan	Industry	Unidirectional - Forward	From the back - Suction
WO2020259599A1 (61)	2020	China	Industry	Unidirectional - Backward	From the back - Suction
WO2021137746A1 (65)	2021	Sweden	Individual Applicant	Multidirectional - Forward & radial	From the side - Macroshape
WO2021117649A1 (39)	2021	Japan	Industry	Unidirectional - Forward	From the back - Suction
WO2021021929A1 (72)	2021	USA	Industry	Multidirectional - Forward & radial<br>Multidirectional - Forward & circumferential	From within - Microshape<br>From within - Macroshape<br>From the side - Friction

WO2021101686A1 (41)	2021	USA	Industry	Unidirectional - Forward	From the side - Friction
WO2021083843A1 (32)	2021	Belgium	Academic Institution	Unidirectional - Forward	From the side - Macroshape
WO2021187916A1 (46)	2021	South Korea	Industry	Unidirectional - Forward	From the back - Suction
WO2022244711A1 (80)	2022	Japan	Industry	Multidirectional - Forward & circumferential	From the side - Friction
WO2022074539A1 (60)	2022	Israel	Industry	Unidirectional - Backward<br>Multidirectional - Forward & circumferential	From the front - Macroshape<br>From the side - Friction<br>From the side - Macroshape
WO2022066464A2 (54)	2022	USA	Industry	Unidirectional - Forward<br>Unidirectional - Backward<br>Multidirectional - Forward & circumferential	From the back - Suction<br>From within - Macroshape
WO2023107250A1 (51)	2023	USA	Individual Applicant	Unidirectional - Forward	From the front - Macroshape
WO2025037297A1 (81)	2025	Israel	Industry	Multidirectional - Forward & circumferential	From the front - Macroshape
WO2025046643A1 (83)	2025	Japan	Industry	Multidirectional - Forward & circumferential	From the back - Suction
WO2025117673A1 (15)	2025	USA	Academic Institution	Unidirectional - Forward	From the front - Macroshape
WO2025119449A1 (82)	2025	Germany	Industry	Multidirectional - Forward & circumferential	From the side - Friction
WO2025074374A1 (16)	2025	India	Industry	Unidirectional - Forward	From the side - Friction
